# Case Report: A homozygous selenocysteine insertion sequence-binding protein 2 (*SECISBP2*) gene mutation in a pediatric patient

**DOI:** 10.3389/fped.2025.1637116

**Published:** 2025-08-22

**Authors:** Lina Almohammadi, Lama Alsayel, Mohammad Aljumaa, Raghad Alhuthil, Afaf Alsagheir

**Affiliations:** ^1^ College of Medicine, Alfaisal University, Riyadh, Saudi Arabia; ^2^ Section of Pediatric Endocrinology, Department of Pediatrics, King Faisal Specialist Hospital and Research Centre, Riyadh, Saudi Arabia

**Keywords:** *SECISBP2* gene, thyroid function, dysmorphic features, short stature, Saudi Arabia

## Abstract

Selenocysteine insertion sequence-binding protein 2 (*SECISBP2*) is crucial for the biosynthesis of selenoproteins, including iodothyronine deiodinases, which play a vital role in thyroid hormone metabolism. Mutations in *SECISBP2* can disrupt thyroid function, leading to various clinical manifestations across multiple systems. We present the case of a 3-year-old Saudi female who was referred for genetic testing due to poor growth, developmental abnormalities, and notable facial dysmorphism. Laboratory tests indicated elevated FT4 levels, low T3 levels, and modestly elevated TSH values. Whole-exome sequencing revealed a homozygous pathogenic variant in the *SECISBP2* gene (c.358C>T; p.Arg120Ter), correlating with the laboratory findings and the patient's clinical presentation. Additional variants of uncertain significance (VUS) in the *ARCN1* and *DNA2* genes were also identified but were considered clinically insignificant due to inheritance patterns and the absence of corresponding phenotypes in heterozygous family members. Treatment with liothyronine (L-T3) led to significant clinical improvement in growth and energy levels over a two-year follow-up period. This case highlights the importance of identifying the specific biochemical profile associated with *SECISBP2* deficiency and advocates for the inclusion of *SECISBP2* in genetic testing panels for endocrine and neurodevelopmental disorders to prevent diagnostic delays. The therapeutic efficacy of liothyronine in such cases is further supported.

## 1 Introduction

The human body expresses 25 selenoproteins, three of which—iodothyronine deiodinases—are essential for thyroid hormone metabolism and the conversion of thyroxine (T4) to triiodothyronine (T3) (1). Selenocysteine insertion sequence-binding protein 2 (*SECISBP2*) is crucial for facilitating the incorporation of selenocysteine into these proteins by recoding the UGA stop codon within the 3′-untranslated region of selenoprotein mRNAs (2–4). Mutations in *SECISBP2* disrupt this process, leading to abnormal selenoprotein production and a range of multisystem manifestations, including growth delay, developmental disabilities, and thyroid hormone abnormalities (2).

Biochemically, *SECISBP2* deficiency is characterized by a distinct thyroid profile: elevated or normal TSH levels, high serum T4, low serum T3, and elevated reverse T3, which result from impaired activity of the three deiodinases (4, 5). The clinical phenotype can be complex, demonstrating variable neurological, skeletal, and endocrine involvement.

This report presents the clinical and molecular findings of a pediatric patient with a homozygous pathogenic *SECISBP2* mutation, emphasizing the diagnostic challenges and therapeutic responses observed. The clinical course highlights the necessity of focusing on *SECISBP2*-related thyroid dysfunction while considering the potential—though not definitive—contributions of coexisting variants.

## 2 Case description

from a local hospital in the Al-Hasa region (eastern Saudi Arabia) in December 2019 for further genetic evaluation due to unexplained growth retardation, developmental delay, and dysmorphological features.

She was born full-term via lower-segment cesarean section following an uneventful pregnancy. The neonatal period was unremarkable, and she was discharged on the same day as her mother. However, developmental concerns became apparent during infancy. She was able to sit independently by 10 months, walked at 1 year and 11 months, and exhibited delays in speech along with mild intellectual disability. Additional concerns included poor weight gain, limited oral intake, and diffuse hair loss.

**Figure 1:**
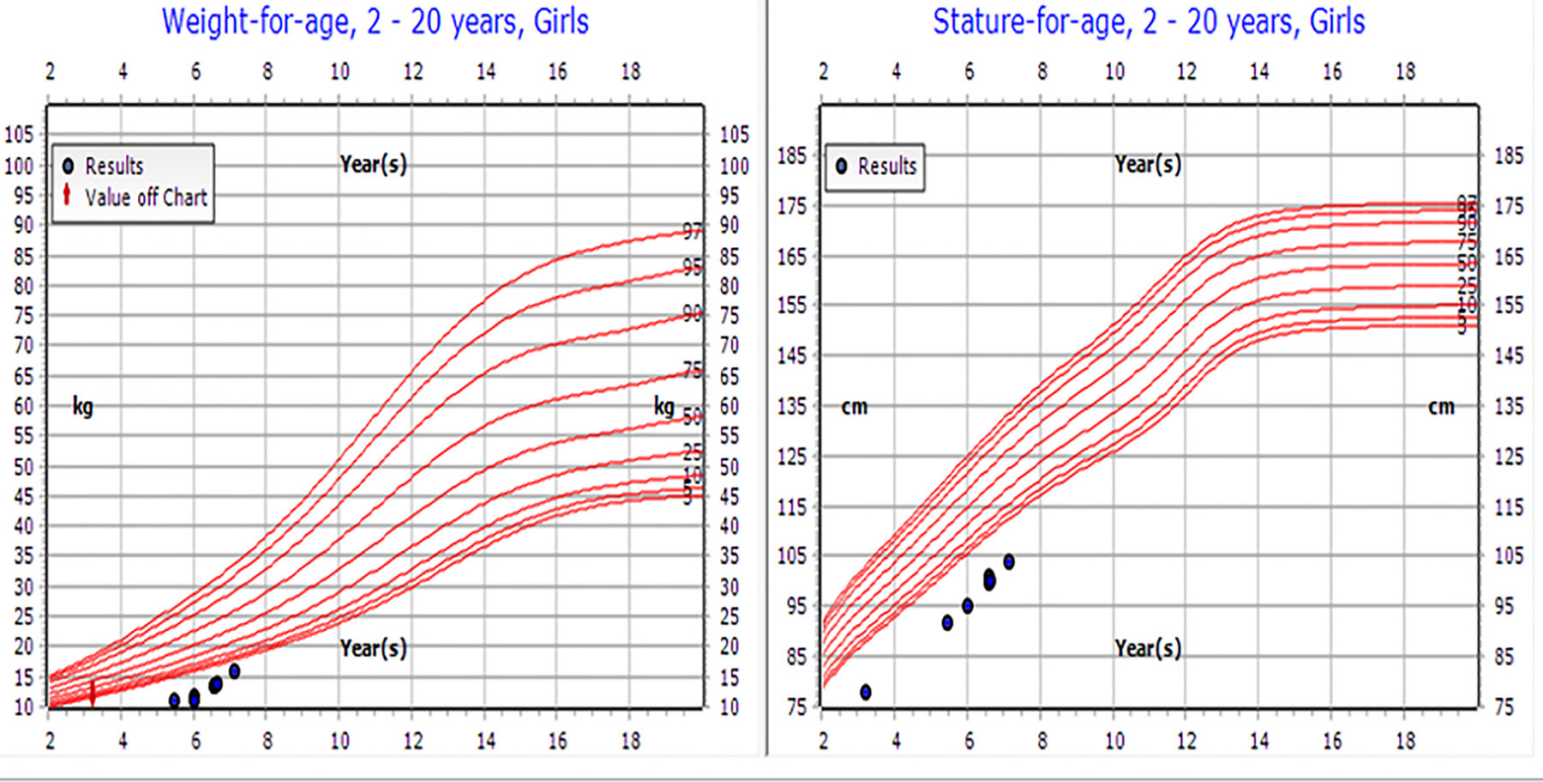
Patient's growth chart.

On physical examination, the patient's weight was 8.4 kg [−5.81 standard deviation scores (SDS)], height was 78 cm (−4.54 SDS), BMI was 13.8 (below the 4th percentile), and head circumference was 38 cm (below the 3rd percentile) (Figure 1). Dysmorphic features included microcephaly, bilateral ptosis, refractive error (for which she wore glasses), large protruding ears, and a small nose, hands, and feet. The examination revealed no gross skeletal anomalies. She was the first child of consanguineous parents (first-degree cousins), both of whom are healthy and medically free. She has a healthy younger brother aged 7 months. The mid-parental height was 162 cm, and the patient's projected adult height fell significantly below this target, further indicating potential pathological growth failure.

### 2.1 Investigation

Initial laboratory testing revealed thyroid function abnormalities, including elevated FT4 levels (33.5 pmol/L; reference range: 12–22), low T3 levels (1.1 nmol/L; reference range: 1.3–3.1), and mildly elevated TSH levels (5.32 mU/L; reference range: 0.27–4.2) (Table 1). These findings were indicative of impaired peripheral conversion of T4 to T3.

**Table 1:** Patient information during follow-up visits.

Visit date	December 2019	March 2022	October 2022	May 2023	November 2023
Age (years)	3	5	6	6	7
Height (cm)	78	91.7	95	100	104
Height (SDS)	−4.54	−4.32	−4.34	−3.92	−3.62
Height GV (cm/year)	N/A	3.8	5.9	8.17	7.74
Weight (kg)	8.4	11	11.6	13.7	16
Weight (SDS)	−5.81	−5.51	−5.57	−4.28	−2.9
Body mass index	13.8	13.1	12.9	13.7	14.8
Ca (ref.: 2.10–2.60) (mmol/L)	2.51	Not done	*H 2.73	*H 2.68	Not done
Po4 (ref.: 1.00–1.80) (mmol/L)	1.38	Not done	*H 1.96	*H 1.82	Not done
FT4 (ref.: 12.0–22.0) (pmol/L)	*H 33.5	*H 36.1	27.3	23.0	18.4
TSH (ref.: 0.270–4.200) (mU/L)	*H 5.320	*H 4.980	*H 4.640	4.13	2.18
T3, total (ref.: 1.3–3.1) (nmol/L)	1.1	1.2	1.7	1.9	2.8
IGF-1 (ref.: 80–244) (ng/ml)	Not done	106	Not done	Not done	Not done
GH, peak (ng/ml)	Not done	9.8	Not done	Not done	Not done
Treatment	None	None	Liothyronine 5 mcg (QD)	Liothyronine 5 mcg (QD)	Liothyronine 5 mcg BID and multivitamins

Ref., reference range; *H, high; *L, low; GV, growth velocity.

**Figure 2:**
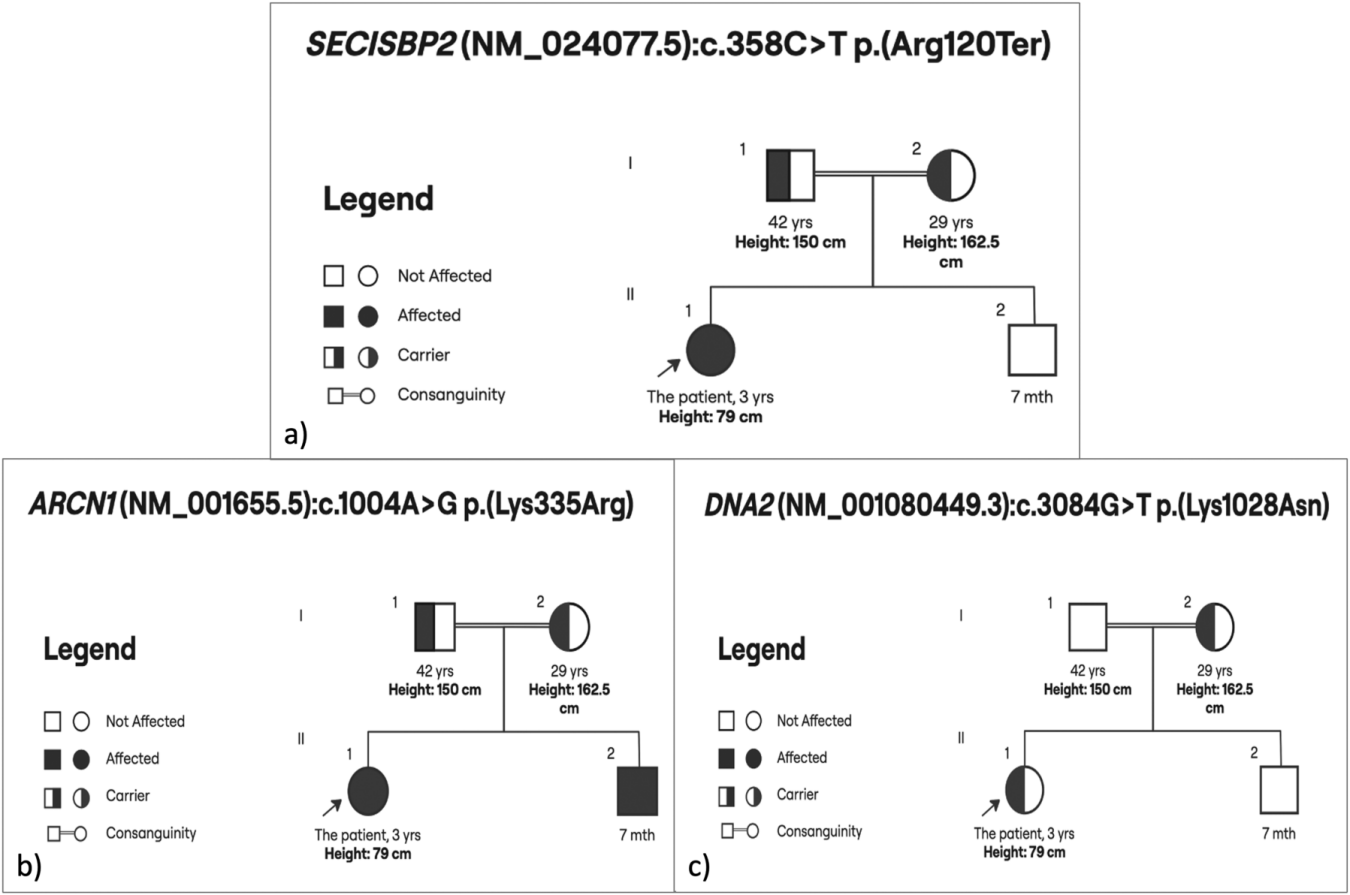
Family pedigrees of the affected patient with identified mutations: **(a)** homozygous *SECISBP2* (NM_024077.5):c.358C>T; p.(Arg120Ter). **(b)** homozygous *ARCN1* (NM_001655.5):c.1004A>G; p.(Lys335Arg). **(c)** heterozygous *DNA2* (NM_001080449.3):c.3084G>T; p.(Lys1028Asn).

Whole-exome sequencing and deletion/duplication analysis were performed on DNA extracted from peripheral blood. A homozygous pathogenic variant in the *SECISBP2* gene (NM_024077.5): c.358C>T; p.(Arg120Ter) was identified. Additionally, a homozygous VUS in the *ARCN1* gene (NM_001655.5): c.1004A>G; p.(Lys335Arg) was detected, along with a heterozygous VUS in the *DNA2* gene (NM_001080449.3): c.3084G>T; p.(Lys1028Asn) (Figure 2 and Table 2).

**Table 2:** Summary of reported genetic variants and predicted impact.

Case	Gene/Mutation (Transcript)	Exon	dbSNP ID	Conservation (phyloP100)	In Silico Prediction	ACMG Classification	Zygosity/Variant Type/Inheritance
1	*SECISBP2* (NM_024077.5): c.358C>T; p.(Arg120Ter)	3	rs777447200	1.156	Not available	Pathogenic (6)	Homozygous/Nonsense/AR
2	*ARCN1* (NM_001655.5): c.1004A>G; p.(Lys335Arg)	7	rs1938940159	8.804	BP4: Benign Supporting (7)	VUS (7)	Homozygous/Missense/AD
3	*DNA2* (NM_001080449.3): c.3084G>T; p.(Lys1028Asn)	20	Not available	1.016	BP4: Benign Supporting (8)	VUS (8)	Heterozygous/Missense/AD

AR, autosomal recessive; AD, autosomal dominant; VUS, variant of uncertain significance; ACMG, American College of Medical Genetics and Genomics; dbSNP, single nucleotide polymorphism database; BP4, ACMG code for computational evidence supporting a benign effect; phyloP100, Phylogenetic *p*-value score based on conservation across 100 vertebrate species.

Segregation analysis indicated that the 7-month-old brother was homozygous for the *ARCN1* variant but exhibited no clinical symptoms. The father was heterozygous for both the *SECISBP2* and *ARCN1* variants, while the mother carried heterozygous variants in *SECISBP2*, *ARCN1*, and *DNA2*. The *DNA2* variant was also identified in the mother in a heterozygous state, and she showed no phenotype consistent with mitochondrial or myopathic disease.

Given the biochemical and clinical phenotype, along with the inheritance pattern, the *SECISBP2* mutation was considered causative. The *ARCN1* and *DNA2* variants did not align with the patient's clinical presentation and are more likely to be benign or only contributory in an oligogenic context.

The patient was lost to in-person follow-up during the COVID-19 pandemic in 2020 and 2021 but continued to be monitored through virtual consultations. At age 5 (March 2022), she presented with fatigue, hypersomnia (sleeping up to 14–16 h), constipation, and recurrent hair loss. Biochemical testing indicated persistent low T3 levels, elevated FT4 and TSH levels, and mildly elevated serum calcium and phosphate levels (Table 1). Her dietary intake included daily milk with cornflakes, which may have affected her mineral levels.

**Figure 3:**
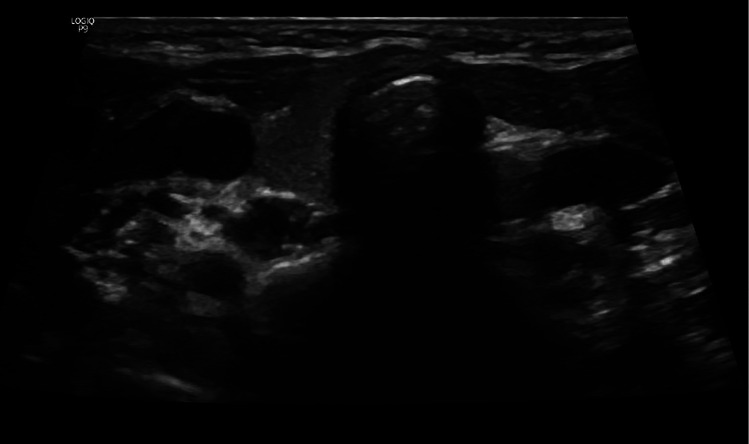
Neck ultrasound showing a non-visualized left thyroid lobe.

**Figure 4:**
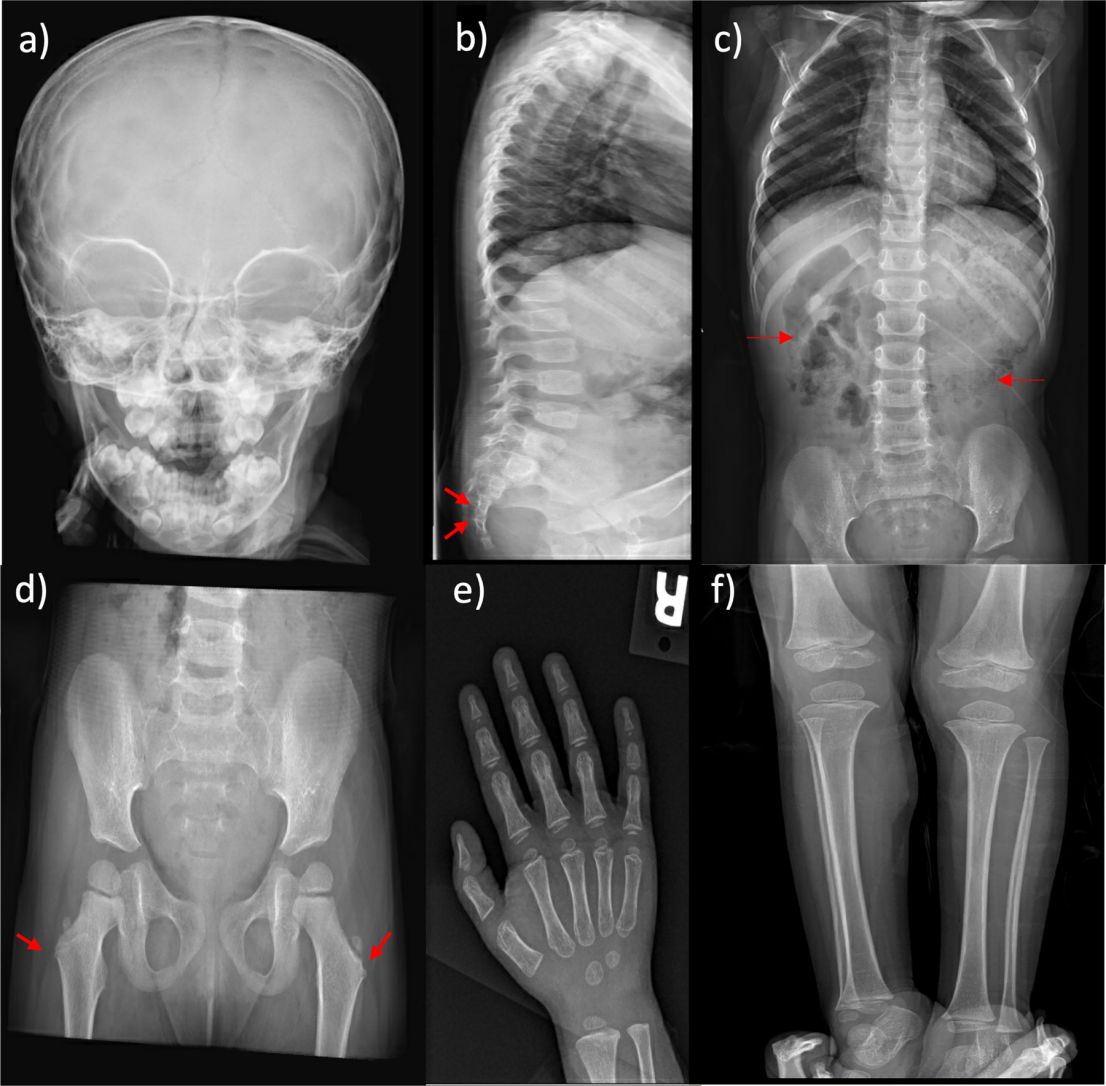
Skeletal survey at 4 years of age. **(a)** The skull exhibits patent sutures and an unremarkable skull base. **(b)** The spine shows more than 50% anterolisthesis of S2 over S3 (arrows). Vertebral alignment, height, and disc spaces in the cervical, thoracic, and lumbar regions are within normal limits, with no segmentation or formation anomalies. **(c)** The abdomen reveals the presence of fecal matter throughout the colon (arrows) and a rounded calcific density over the right 11th rib on the anteroposterior view. **(d)** The pelvis exhibits mild bilateral coxa valga (arrows), whereas the rest of the bony pelvis is unremarkable. **(e,f)** Upper and lower extremities show no skeletal dysmorphic features.

A neck ultrasound revealed a non-visualized left thyroid lobe (Figure 3). A pituitary MRI was conducted to exclude central causes of growth failure and thyroid dysfunction; the imaging results were unremarkable. A dysmorphic skeletal survey showed anterolisthesis of S2 over S3 (Figure 4), but no other significant abnormalities were noted. Bone age, assessed through a hand radiograph, was estimated at 28 months, despite her chronological age of 5 years, indicating delayed skeletal maturation.

### 2.2 Treatment

The patient was started on liothyronine (Cytomel) at a dosage of 5 mcg once daily (QD) to address her persistent low T3 levels and normalize thyroid function. Over the following months, there was significant improvement in clinical symptoms, including constipation, fatigue, and hair loss. The dosage was subsequently increased to 5 mcg twice daily (BID) for maintenance. Additionally, a multivitamin supplement was prescribed to help prevent potential micronutrient deficiencies.

### 2.3 Outcome and follow-up

At the most recent follow-up in November 2023, the patient, now 7 years old, demonstrated significant clinical improvement. Her weight increased from −5.81 to −2.9 SDS, and her height improved from −4.54 to −3.62 SDS. Her annual growth velocity reached 8 cm per year. Although her bone age remained delayed, the enhancements in height and energy levels indicate a positive response to liothyronine therapy (see Table 1 and Figure 1). Moreover, her cognitive and speech development has remained stable.

## 3 Discussion

This report presents a complex genetic case involving a homozygous pathogenic mutation in the *SECISBP2* gene (6), along with two additional VUS in the *ARCN1* (7) and *DNA2* genes (8). Among these, the *SECISBP2* variant (c.358C>T; p.Arg120Ter) provides the most compelling molecular explanation for the patient's biochemical and clinical phenotype. *SECISBP2* is essential for selenoprotein synthesis, and its disruption impairs the function of deiodinases necessary for thyroid hormone metabolism. This results in a characteristic biochemical profile that includes elevated or normal TSH, elevated T4, low T3, and elevated reverse T3 (1–3). Clinical manifestations can include growth failure, delayed bone maturation, neurodevelopmental delays, and dysmorphic features.

Although the prevalence of *SECISBP2* mutations is currently unknown, they have been reported across various populations (2, 5, 9–14). The homozygous nonsense mutation identified in our patient, c.358C>T (p.Arg120Ter), is currently considered a pathogenic variant (14). This same mutation was observed in a Brazilian girl with delayed bone age, congenital myopathy, and neurological impairment who carried compound heterozygous *SECISBP2* mutations (11). Another case described a girl of Guinean descent who presented with hypotonia and feeding difficulties while carrying the same homozygous variant (12). In both instances, the biochemical profiles were similar, and while L-T3 therapy improved some systemic manifestations, neurocognitive deficits persisted.

Other reported genotypes, such as a homozygous c.382C>T (p.Arg128Ter) mutation in an African boy, presented only with short stature and delayed bone age, despite being clinically euthyroid. This suggests phenotypic variability that may be linked to alternative start codons or residual protein activity (13). Moreover, Stoupa et al. emphasized the heterogeneity of *SECISBP2* presentations, which can include absence of speech, seizures, and features of autism spectrum disorder across six unrelated families. Some patients experienced misdiagnosis or inappropriate treatment due to misinterpretation of thyroid function tests and the exclusion of *SECISBP2* from neurodevelopmental gene panels (12). These findings underscore the critical importance of early recognition and comprehensive genetic screening in children who present with multisystem symptoms and atypical thyroid function profiles.

In addition to the pathogenic *SECISBP2* mutation, our patient carried a homozygous missense variant in *ARCN1* (c.1004A>G; p.Lys335Arg) and a heterozygous missense variant in *DNA2* (c.3084G>T; p.Lys1028Asn). While mutations in *ARCN1* have been associated with craniofacial dysmorphism and developmental delay (15), and *DNA2* variants with mitochondrial myopathy and external ophthalmoplegia (16, 17), neither variant was deemed causative in this case due to several reasons. The *ARCN1* variant, for instance, was found to be homozygous in the patient's phenotypically normal 7-month-old brother, while the heterozygous *DNA2* variant was inherited from the asymptomatic mother. Additionally, both variants are missense mutations that had not been previously reported as pathogenic in population or clinical databases, and in silico prediction tools classified them as benign (6, 7).

Taken together with their autosomal dominant inheritance patterns, these findings suggest limited clinical relevance of the *ARCN1* and *DNA2* variants in this case. However, the potential for an oligogenic contribution cannot be entirely ruled out. Interactions between genes may influence the severity or range of *SECISBP2*-related phenotypes, which is a possibility that warrants further investigation.

Regarding management, there are currently no standardized treatment guidelines for *SECISBP2* deficiency. Our patient demonstrated significant clinical improvement in both growth and energy levels following treatment with liothyronine (L-T3). While bone age remained delayed, there was evidence of catch-up growth, and thyroid hormone levels gradually returned to normal. These findings support previous research indicating that L-T3 can alleviate symptoms, although neurodevelopmental progress may be limited if treatment is started late. It is essential to include SECISBP2 in gene panels for thyroid dysfunction and developmental delay to avoid diagnostic delays and inappropriate treatment.

In conclusion, this case illustrates the value of considering *SECISBP2* deficiency in pediatric patients who present with growth retardation and abnormal thyroid function tests. While additional genetic variants may be identified through whole-exome sequencing, their interpretation should take into account the clinical context, family segregation, population frequency, and in silico predictions. Timely diagnosis and personalized management can lead to significantly improved outcomes for affected individuals.

## Data Availability

The raw data supporting the conclusions of this article will be made available by the authors, without undue reservation.
